# Extracellular Vesicles Derived from Trypanosomatids: The Key to Decoding Host–Parasite Communication

**DOI:** 10.3390/ijms26094302

**Published:** 2025-05-01

**Authors:** Armanda Rodrigues, Juliana Inês Weber, João Durães-Oliveira, Cláudia Moreno, Micheli Ferla, Maria de Aires Pereira, Ana Valério-Bolas, Bruna Eugênia de Freitas, Telmo Nunes, Wilson T. Antunes, Graça Alexandre-Pires, Isabel Pereira da Fonseca, Gabriela M. Santos-Gomes

**Affiliations:** 1 Global Health and Tropical Medicine, GHTM, Associate Laboratory in Translation and Innovation Towards Global Health, LA-REAL, Instituto de Higiene e Medicina Tropical, IHMT, Universidade NOVA de Lisboa, UNL, 1349-008 Lisboa, Portugal; a21001039@ihmt.unl.pt (J.I.W.); joaodo@ihmt.unl.pt (J.D.-O.); moreno.claudia@ihmt.unl.pt (C.M.); a21002275@ihmt.unl.pt (M.F.); mapereira@esav.ipv.pt (M.d.A.P.); ana.bolas@ihmt.unl.pt (A.V.-B.); brunaeugenia@hotmail.com (B.E.d.F.); santosgomes@ihmt.unl.pt (G.M.S.-G.); 2 CERNAS-IPV Research Centre, Instituto Politécnico de Viseu, Campus Politécnico, Repeses, 3504-510 Viseu, Portugal; 3 Instituto de Ciências Biológicas, ICB, Universidade Federal de Minas Gerais, Belo Horizonte 31270-901, MG, Brazil; 4 Microscopy Center, Faculty of Sciences, University of Lisbon-FCUL-BioISI Ce3CE, 1749-016 Lisboa, Portugal; telmonunes@hotmail.com; 5 Instituto Universitário Militar (IUM), Centro de Investigação, Desenvolvimento e Inovação da Academia Militar (CINAMIL), Unidade Militar Laboratorial de Defesa Biológica e Química (UMLDBQ), 1849-012 Lisboa, Portugal; antunez.wdta@gmail.com; 6 CIISA, Centre for Interdisciplinary Research in Animal Health, Faculty of Veterinary Medicine, University of Lisbon, 1649-004 Lisbon, Portugal; gpires@fmv.ulisboa.pt (G.A.-P.); isabelfonseca@fmv.ulisboa.pt (I.P.d.F.); 7 Associate Laboratory for Animal and Veterinary Sciences (AL4AnimalS), 1200-771 Lisbon, Portugal

**Keywords:** extracellular vesicles, trypanosomatids, immunomodulation, host–parasite interactions, biomarkers, diagnostic, vaccines

## Abstract

Trypanosomatids constitute a family of parasitic protozoa that cause significant human and veterinary diseases that are classified as neglected zoonotic diseases (NZDs). In a rapidly evolving world, these diseases have the potential to become a world health problem no longer solely associated with low-income countries. Therefore, the development of new strategies to control and restrain the dissemination of trypanosomatids is imperative. Extracellular vesicles (EVs) are a heterogeneous group of membrane-enclosed vesicles released by prokaryotic and eukaryotic cells. They can be found in diverse body fluids that carry biologically active molecules, including proteins, nucleic acids, lipids, and carbohydrates. EVs participate in cell-to-cell communication by delivering their cargo content to recipient cells. Thus, EVs play a role in regulating normal physiological processes, including immune surveillance and tissue repair, as well as being involved in pathological conditions, like cancer. In recent years, EVs have attracted significant attention from the scientific community, mainly due to their immune regulatory properties. Therefore, this review examines the role played by trypanosomatid-derived EVs in leishmaniases and trypanosomiasis, highlighting their biological role in host–parasite communication and exploring their potential future applications in controlling NZDs, especially those caused by trypanosomatids.

## 1. Introduction

Neglected tropical diseases (NTDs) are defined as a group of infectious diseases, which are mainly chronic, debilitating, often stigmatizing, and associated with extreme poverty in remote rural areas, urban slums, and conflict zones in Africa, Asia, and Latin America [1]. However, as the world is rapidly evolving, these diseases have the potential to become a global health problem that extends beyond low-income populations. According to the World Health Organization, there are 17 NTDs, 3 of which are directly caused by trypanosomatids: Chagas disease (American trypanosomiasis), sleep sickness (human African trypanosomiasis), and leishmaniasis. These are also classified as neglected zoonotic diseases (NZDs) due to their zoonotic character [2]. Given that zoonoses are diseases transmitted from vertebrate animals to humans, their management requires integrated approaches that address both human and animal health.

Trypanosomatids are a family of protozoa within the order Trypanosomatida, characterized by having a single flagellum. *Trypanosoma* spp. and *Leishmania* spp. are necessarily dixenic species that have zoonotic or anthroponotic life cycles and are transmitted by blood-sucking insects [3,4,5,6,7,8,9]. The tsetse fly (genus: *Glossina*) and insects of the subfamily *Triatominae* are the natural vectors of *Trypanosoma brucei* and *Trypanosoma cruzi*, respectively [9]. In turn, female sand flies (Diptera: *Psychodidae*: Phlebotominae) constitute the natural vectors of *Leishmania* spp. [10,11]. A total of 2 subspecies of *T. brucei* (*T. brucei gambiense* and *T. brucei rhodesiense*), *T. cruzi*, and 21 species of *Leishmania* are pathogenic to humans, causing significant disease. Moreover, in addition to their impact on human health, leishmaniasis caused by *L. infantum*, and trypanosomiasis caused by *T. cruzi*, *T. evansi*, *T. brucei brucei*, *T. vivax*, *T. simiae*, *T. suis*, and, more rarely, *T. godfreyi*, can also affect domestic animals. These parasites have a substantial economic impact on different productive livestock systems, especially in Africa and South America, as cattle, sheep, goats, horses, and donkeys are susceptible to infection [12,13,14,15,16,17,18]. Overall, the losses of livestock production across sub-Saharan Africa have been estimated to reach up to 20% [19].

Despite the efforts to control or eradicate NZDs, the estimated number of new cases of human infection remains significant. Approximately 30,000 new cases of visceral leishmaniasis (VL) and more than 1 million new cases of cutaneous leishmaniasis (CL) occur annually, and 6 to 7 million people worldwide are currently infected with *T. cruzi* [1]. In contrast, the number of African trypanosomiasis cases had declined to fewer than 1000 new cases per year [20]. However, all these conditions require medical treatment, and some are life-threatening diseases. The emergence of the SARS-CoV-2 pandemic along with the efforts to contain it had an overwhelming impact on the fragile health systems and NTD/NZD control programs among the low-income countries where these diseases are endemic [21]. Moreover, NZDs are no longer only associated with low-income countries. The multifaceted nature of human–animal relations is subject to constant adjustment, influenced by climate change and anthropogenic and natural factors. The increase in travel, tourism, and the international trade of live animals, as livestock species and pets or as part of breeding programs for endangered wildlife species, constitutes a significant role in shaping the epidemiology of NZDs and poses important challenges to global health [22,23]. Therefore, the development of new strategies to control and restrain the dissemination of diseases caused by trypanosomatids are urgent.

As in most infections, it is the interaction of the parasite–host immune system that constitutes a decisive point for infection outcome. The balance between parasite survival and the host’s immune system dictates the infection outcome, which can result in parasite elimination or parasite survival within the host, thereby ensuring the completion of its life cycle [24]. Consequently, trypanosomatids have evolved strategies to persist and disperse inside the host by evading, invading, subverting, and exhausting host immune defenses, including cell memory [24,25]. It has been demonstrated that the host immune response is mainly organ-specific, as in canine leishmaniasis (CanL) [25,26]. The mammalian immune system consists of two types of immune responses: innate response and specific or acquired response. Together, these two immune responses are responsible for the recognition of pathogens and their consequent inactivation and elimination [27]. After the initial contact with a pathogen, the mammalian immune system can also acquire immune memory that allows for faster and more effective immune protection in subsequent contact with the same pathogen (or with the same antigen) [28]. The innate immune response constitutes the host’s first line of defense, which means that, in the presence of a pathogen, its function is essential for the recognition and early triggering of an inflammatory response. Unlike the innate response, the acquired immune response is specific to the pathogen and is mediated by T and B lymphocytes [29,30]. Parasites promote their survival and establish infection in the host by influencing the immune system through cell-to-cell communication. The proteins and vesicles (secretome) derived from parasites appear to play a decisive role in parasite–host interactions by promoting host immunomodulation, favoring the parasite’s survival [24,25].

The present review focuses on the extracellular vesicles (EVs) generated by trypanosomatids (TEVs) during their life cycle and how these naturally released vesicles can influence the host immune response. For this, combined data on human and animal trypanosomiasis were analyzed, constituting an integrative and comprehensive approach that brings together the most up-to-date and groundbreaking findings in the field. It also highlights the potential applications of EVs as diagnostic biomarkers and vaccine candidates and explores their role as host immunomodulators.

## 2. Key Challenges in the Study of Trypanosomatid-Derived EVs

Extracellular vesicles are a heterogeneous group of membrane-enclosed vesicles that do not replicate. They are naturally released by prokaryotic and eukaryotic cells and can be found in diverse body fluids, including blood, urine, saliva, breast milk, amniotic fluid, ascites, cerebrospinal fluid, bile, and semen. EVs have been found to contain biologically active molecules, including proteins, nucleic acids, lipids, and carbohydrates [31,32]. These sac-like structures are of cellular origin and may carry not only DNA and mRNA but also small nucleolar RNA (snRNA), Y RNA, mitochondrial RNA, a variety of small untranslated RNA molecules (vault RNA), and long non-coding RNA, whose presence has only recently been acknowledged [33,34,35]. EVs have been shown to participate in cell-to-cell communication by transferring their cargo content to recipient cells [32,36,37] and have been implicated in the regulation of physiological conditions, such as immune surveillance and tissue repair, as well as in diverse pathological conditions [32], including diseases caused by protozoa [38,39,40,41].

Historically, EVs have been classified into three different categories based on their biogenesis pathway and size (exosomes, microvesicles, and apoptotic bodies) [42,43], which are commonly employed in most of the publications on EVs. Exosomes have been described as small vesicles (30 to 100–150 nm) of endosomal origin [44,45]. For cargo transfer, exosomes fuse with the plasma membrane of the recipient cell or are internalized by endocytosis or phagocytosis [46,47]. In contrast to exosomes, microvesicles are shed directly from the plasma membrane and can exhibit a highly variable size, ranging from 0.1 to 2 μm [32]. Although generated by different biogenesis pathways, it is not always possible to differentiate between exosomes and microvesicles, as their sizes can overlap. Therefore, size is not a reliable criterion, and several studies use the term “exosomes” when referring to a mixed population of EVs [32,48,49]. Unlike exosomes and microvesicles, which are constantly generated by viable and activated cells, apoptotic bodies are secreted as blebs by cells undergoing apoptosis. These vesicles vary in size from 50 to 5000 nm, being the largest vesicle in comparison with other types of EVs [42,50].

Unfortunately, the majority of studies do not fully discriminate between different categories of EVs, as the sizes between EV classes can overlap, and the main differences between EV types lie in their biogenesis [31,32,51,52]. Additionally, there is a lack of consensus among researchers concerning EVs’ nomenclature and specific markers for endosomal origin (exosomes) and plasma membrane source (microvesicles), making the comparison and characterization of isolates particularly challenging. Acknowledging these issues, in 2018, the International Society for Extracellular Vesicles (ISEV) updated the Minimal Information for Studies of Extracellular Vesicles (MISEV) guidelines [53], laying the foundation for the standardization of procedures for EV isolation and adjusting EV nomenclature. It is therefore recommended to adopt a more functional and descriptive system to classify EVs, mainly based on size, molecular signature, and cellular origin.

**Figure 1:**
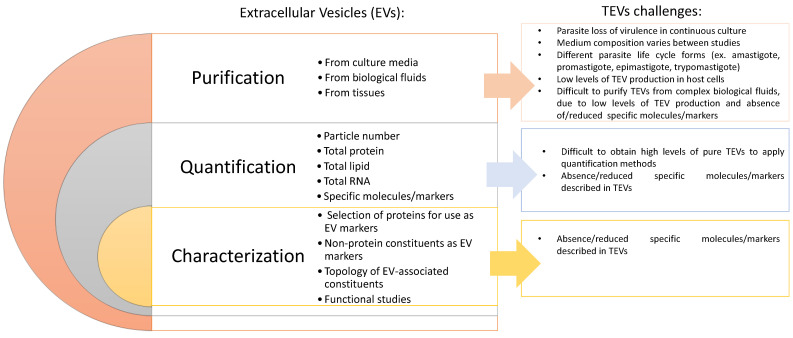
Challenges in EV studies. The left panel illustrates the most critical points of dealing with EVs, according to MISEV2018 actualization guidelines. The right boxes highlight the main challenges in studying trypanosomatid-derived EVs (TEVs).

Although all types of cells release EVs, most of the available research has been focused on mammalian EVs, primarily those of human or mouse origin, with limited EV studies from other cell types or under different experimental conditions. Therefore, the application of these guidelines to the study of TEVs is still backward and full of challenges (Figure 1). Thus, the major current challenges in working with TEVs are (i) the standardization of isolation and purification methodologies, accompanied by (ii) the uniformization of the characterization of TEV molecular markers, establishing a clear profile for each class of TEVs, as well as continuous (iii) innovation in isolation and purification methodologies to allow for a clear discrimination between the different categories of TEVs. Altogether, this will facilitate a comparison across published studies, as well as promoting advances in the field of TEV research, its biological applications, and future clinical translation.

## 3. Composition of EVs Derived from Trypanosomatids

*Leishmania* spp. and *Trypanosoma* spp. have complex life cycles, requiring rapid adaptation to changing environments from the mammalian host to the hematophagous vector in order to survive. Therefore, the development of various strategies to respond to these changes and persist in the host was evolutionarily selected [32]. Although EVs may represent a primordial form of cell-to-cell communication, preceding the appearance of a more specific intercellular crosstalk involving ligands and receptors [54], they are effective mediators of cellular communication among diverse interacting organisms, such as mammals, pathogens, and arthropod vectors. This inter-kingdom communication via EVs is likely to have a crucial evolutionary impact by influencing parasite adaptation to specialized niches in the host, driving host resistance and evolutionary responses, along with the maintenance of parasite virulence and transmissibility [30]. The release of TEVs throughout the life cycle of trypanosomatids represents a fundamental component of parasitic infection. They play a prominent role in parasite–host interaction by manipulating the host’s physiology and modulating host immune responses, facilitating the spreading of virulence factors [32,55,56], and influencing the social motility of parasites [57].

### 3.1. Leishmania-Derived EVs

*Leishmania* parasites deal with two distinct environments, the sand fly midgut and the host macrophage (MØ) phagolysosome, which requires the expression of stage-specific virulence factors. *Leishmania*-derived proteins (exoproteome), including virulence factors, can be released from the surface of the parasite through either a classical or nonclassical mechanism of secretion (secretome) [58]. A proteomic analysis of the exoproteome of *L. donovani* revealed that only 14% of the detected proteins contain N-terminal signal sequences, which play a role in targeting the protein to specific organelles or cell secretion. In contrast, the remaining proteins used nonclassical mechanisms of secretion [59]. Thus, it has been proposed that the nonclassical mechanisms of secretion represent one of the strategies employed by *Leishman*ia parasites to communicate with the host environment, thereby ensuring successful infection [60].

**Figure 2:**
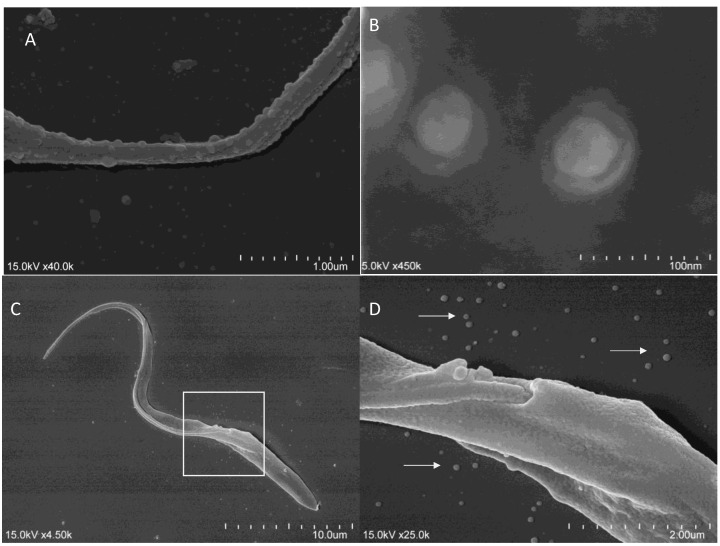
Scanning electron microscopy images of trypanosomatids and TEVs. *L infantum* flagellum exhibiting EV budding (**A**); purified EVs from *L. infantum* promastigotes (**B**). *T. cruzi* epimastigote (**C**) and a magnified view of the white square (**D**). White arrows indicate epimastigote-derived EVs. Parasites were grown in Schneider’s Drosophila medium (SCH, Sigma–Aldrich, St. Louis, MO, USA) supplemented with exosome-depleted fetal bovine serum (exo-free FBS, Gibco™, Thermo Fisher Scientific, Waltham, MA, USA), and digital images were obtained by the authors under an ultra-high-resolution scanning electron microscope (SU8010 Hitachi, Tokyo, Japan).

*Leishmania* parasites alternate between an extracellular life stage (promastigote) and an intracellular life stage (amastigote). Thus, promastigote forms can secrete EVs into the extracellular space, while amastigotes can change the composition of their host cells through EVs. Silverman and colleagues [59] described, for the first time, the secretion of EVs by *L. donovani* promastigotes cultured in a conditioned medium. The presence of EVs was observed at the promastigote flagellar pocket, as well as across the surface of amastigotes undergoing axenic differentiation. Furthermore, it has been reported that EVs have been isolated from a conditioned cultured medium of several species of *Leishmania*, including *L. major* [61,62], *L. donovani* [61], *L. mexicana* [61,63,64], *L. braziliensis* [56], *L. infantum* [60,65], *L. amazonensis* [66], *L. shawi*, and *L. guyanensis* [67] (Figure 2A,B).

**Figure 3:**
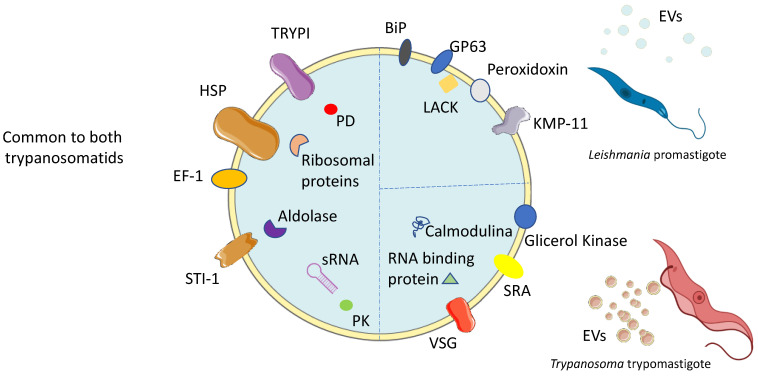
A schematic representation of the molecular signature of TEVs. Common proteins and nucleic acids found in TEVs, such as several heat shock proteins (HSPs), small RNA (sRNA), elongation factor 1 (EF-1), aldolase, various ribosomal proteins, several peptidases and kinases, and tryparedoxin peroxidase (TRYPI), among others, are shown. Molecular components specific to *Leishmania*- or *Trypanosoma*-derived EVs are indicated.

The advancement in high-throughput techniques has enabled the execution of multiple EV studies based on molecular cargo profiles. Some studies have characterized EVs secreted by *Leishmania* promastigotes and axenic amastigotes based on their diameter, morphology, density, and protein cargo, such as the glycoprotein of 63 kDa (GP63), heat shock protein (HSP) 70, HSP90, calpain-like cysteine peptidase, carboxypeptidase, S-adenosylmethionine synthase and elongation factor 1 (EF-1), and acetylcholinesterase activity [60,61,63,64]. Although several studies have characterized the exoproteome of *Leishmania* spp. [59,63], few have specifically examined the cargo content of EVs [65,68]. A proteomic analysis of EVs purified from culture-conditioned medium allowed for the identification of 329 proteins, which account for 52% of the total proteins secreted by *L. donovani.* This finding reinforces the importance of EV-based secretion as a key mechanism for protein export (Figure 3). Interestingly, when subjected to high temperature, simulating promastigote transference from the sand fly vector (25 °C) to the mammalian host (37 °C), *L. donovani* axenic promastigotes upregulate EV release. Furthermore, when cultured under low-pH conditions to ensure a full differentiation into amastigotes, therefore mimicking phagolysosome maturation, the protein cargo of EVs changes. The increase in EV secretion and the alteration in EV cargo may represent a sophisticated mechanism for the delivery of effector molecules (virulence factors) in response to specific environmental conditions, imitating mammalian early infection, to subvert host effector functions. Indeed, several virulence factors were identified in EVs derived from *L. donovani* parasites grown at 37 °C, namely the metalloprotease GP63, HSP 10, HSP 70, tryparedoxin peroxidase (TRYPI), 14-3-3-like protein, kinetoplastid membrane protein-11, activated protein kinase C receptor (LACK), and stress-induced protein 1 (STI 1). On the other hand, HSP 10, TRYPI, and 14-3-3-like protein have been found to be enriched in EVs from axenic amastigotes that replicate in acidic conditions [69]. Thus, EV-based secretion can play a central role in protein export, facilitating the successful establishment of infection.

Under the same in vitro infection-like stressors (acidic pH and elevated temperature), which drive promastigote to amastigote differentiation, EVs purified from supernatants of *L. donovani* or *L. braziliensis* parasites were found to be highly enriched in small non-coding RNAs, particularly tRNA-derived small RNAs. On the other hand, the cargo of EVs secreted by promastigotes cultured at 25 °C, mimicking the conditions faced by the parasite in the vector, was also investigated. In such conditions, *L. chagasi* procyclic and metacyclic promastigotes released EVs containing approximately 50 virulence factors, including GP63, EF1α, oligopeptidase, casein kinase, KMP-11, cysteine peptidase, BiP, and peroxidoxin [68], which are probably involved in parasite establishment within the sand fly. GP63 was also found in EVs released by *L. mexicana* amastigotes [64] and in stationary-phase cultures of *L. major* promastigotes [62], suggesting its role in mammal infection. Despite their protein cargo, EVs have been shown to be carriers of nucleic acids, mainly RNA. Most RNA sequences carried by *Leishmania*-derived EVs were found to originate from non-coding RNA types, including rRNA and tRNA [56,69].

### 3.2. Trypanosoma-Derived EVs

Similar to *Leishmania* spp., *Trypanosoma* is a dixenous parasite that infects vertebrates, including humans, and is transmitted by hematophagous invertebrates. It exhibits complex evolutionary forms, being either an obligatory intracellular (*T. cruzi*) or exclusively extracellular parasite (*T. brucei*). Recent studies have disclosed the sophisticated mechanism used by *Trypanosoma* to communicate with their mammalian hosts through EVs [70]. Szempruch and colleagues [71] reported for the first time the dynamics of EV biogenesis originating from the nanotubes that bud from the trypanosome flagellar membrane followed by vesiculation. However, a recent study in *T. evansi*, the etiological agent of trypanosomiasis in equids, indicated that Ca^+2^ is not involved in the vesiculation process [72].

Significant advances have been made in identifying and characterizing released pathogen EVs in the extracellular environment. Since then, it has been proven that *T. cruzi* produces EVs in different evolutionary stages [55]. Most studies utilize EVs derived from epimastigotes (Figure 2C,D) due to their ability to grow in axenic culture. The complex biology of this parasite makes studying EVs derived from intracellular amastigotes challenging, as their manipulation and purification in substantial quantities remain technically demanding. As for EVs derived from trypomastigote forms, it may be complicated to overcome the contamination with cellular and genomic material from the host. However, significant scientific advances have been made in characterizing the content of *T. cruzi* EVs in distinct environments, including the hematophagous vector or the mammalian host, where intracellular amastigotes develop [73]. A secretome analysis of infective and non-infective forms of *T*. *cruzi* identified a set of proteins involved in metabolism, signaling, and nucleic acid binding, along with factors of virulence. EVs from epimastigotes and metacyclic trypomastigote forms contain mucin-like surface glycoproteins GP35/50, which are typical of the insect stages of the parasite. Furthermore, alternative flagellum calcium-binding protein (FCaBP), which is associated with the modulation of several other proteins through Ca^2+^ signaling, was identified in epimastigote-derived EVs [74].

Among extracellular parasites, a comparative analysis of the secretome of *T. brucei* subspecies (*T. brucei rhodesiense,* which infects humans and animals, and *T. brucei brucei* which does not infect humans) revealed the presence of 50–100 nm EVs budding from the plasma membrane along with several associated proteins [69]. Moreover, proteomic analysis identified 156 proteins across diverse functional classes, of which several were confirmed by immunoblotting. *T. brucei*-derived EVs carry variant surface glycoproteins (VSG), HSP-70, glycerol kinase, aldolase, and ribosomal proteins [61] (Figure 3). They also carry several virulence proteins, including the serum-resistance-associated (SRA) protein, which is an essential virulence factor that can inactivate the host circulating trypanosome lytic factors [71]. Interestingly, this study demonstrated that during the co-infection of the vector (*Glossina* spp.) with the two subspecies, *T. brucei rhodesiense* and *T. brucei brucei,* the SRA protein could be transferred among parasites, improving their ability to infect the host.

A proteomic analysis of *T. cruzi*-derived EVs has revealed the presence of trans-sialidases, mucin-like proteins, and members of the retrotransposon hot spot protein family [75]. Moreover, the glycoprotein Tc85/GP85, which is a key member of the trans-sialidases family, has been described as the predominant component carried by EVs [76]. Furthermore, Seco-Hidalgo and colleagues demonstrated that other components, such as Trypomastigote Alanine-, Valine-, and Serine-rich proteins (TcTASV-C) are abundantly present in EVs released by trypomastigotes across different discrete typing units (DTUs I, II, and VI) [77]. Beyond proteins, *T. cruzi* EVs also carry nucleic acids. Under nutrient starvation conditions, epimastigotes release EVs enriched in small tRNAs and TcPIWI-tryp proteins.

Overall, EVs of different trypanosomatids display distinct proteomic and RNA profiles, highlighting their potential role in regulating disease severity. Nevertheless, the impact of small RNAs on disease outcomes remains largely unexplored, and significant insights into identifying and characterizing RNAs carried by EVs derived from protozoa will improve the understanding of parasite–host interactions. These advances will shed light on the biological functions of RNAs delivered by EVs, opening new avenues for developing innovative prophylactic and therapeutic strategies.

## 4. TEVs as Key Players in Host Immunomodulation

In the host, EVs play a crucial role in intracellular communication as trypanosomatids’ virulence factors can be delivered via TEVs. These factors potentially influence the host cells beyond simple diffusion [78,79,80], modulating the host immune response, as observed with trypomastigote-derived EVs that prime host cells to facilitate parasite invasion [81,82].

*Leishmania* spp. and some species of *Trypanosoma* are intracellular parasites that target host cells with immune functions, as is the case of MØs. Thus, an adequate and well-balanced immune response is essential to control infection and promote a favorable infection outcome for the host. Protective immunity against these intracellular trypanosomatids is classically associated with an inflammatory response and the production of interleukin (IL)-12, tumor necrosis factor (TNF)-α, and interferon (IFN)-γ [83]. Consequently, EVs released from parasite-infected cells can modulate both the innate and acquired immune responses, downregulating the host’s immune response against the parasite, promoting its survival in the host [84]. Parasites can modulate the infected host to secrete cytokines and chemokines along with the expression of their own virulence factors, like metalloproteases and cysteine proteases, proteophosphoglycans (PPGs), lipophosphoglycan (LPS), secreted acid phosphatases (SAPs), and peroxiredoxins, to ensure a permissive environment for their survival and development [49].

### 4.1. Host Immunomodulation by Leishmania-Derived EVs

EVs shed by *Leishmania* promastigotes can also induce host immune mediators. Silverman and collaborators [38] demonstrated that pretreating monocytes with *L. donovani* EVs effectively mimics the parasite infection by inhibiting the production of IL-8 and TNF-α while significantly increasing the production of IL-10 and IFN-γ. Another study has shown that the previous treatment of mice with EVs from *L. donovani* or *L. major* caused an increase in the progression of the infection, leading to the development of disease [85]. These findings suggest that EVs have an immunosuppressive effect on host cell activity.

GP63 is the most abundant surface glycoprotein of *Leishmania* spp. and one of the most important virulence factors [49]. Inside MØs, GP63 cleaves multiple substrates that result in the modulation of host signaling pathways, including the mammalian target of rapamycin (mTOR), protein tyrosine phosphatases (PTPs), and transcription factors (TFs), ultimately favoring parasite survival [86,87]. *L. mexicana*-derived EVs promoted MØ inhibitory factors by activating PTPs and inhibiting the synthesis of nitric oxide (NO), generating a favorable environment for parasite replication [63]. Then, Barbosa and colleagues [88] showed that EVs secreted by *L. amazonensis* favor disease progression by inducing murine MØs and B-1 cells (that rapidly produce low-reactive antibodies of the IgM class) to produce anti-inflammatory IL-4, IL-10, and TNF-α. This, in turn, facilitates an increase in parasite burden and consequently favors lesion development. On the other hand, EVs released from *L. donovani*-infected MØs that also carry GP63 can proteolyze Dicer1, which is involved in the biogenesis of microRNA (miRNA). This cleavage blocks the generation of miRNA-122, which influences MØ activity toward an anti-inflammatory state, thereby facilitating parasite immune evasion and promoting disease exacerbation [79].

Since EVs derived from *Leishmania* spp. enhance host cell infectivity [89], the co-injection of *L. amazonensis* promastigotes with EVs leads to increased inflammation and a higher parasite burden in the footpad of mice [90]. Silverman and coworkers [61] evaluated the exposure of *Leishmania* parasites to the THP-1 monocyte-derived MØ cell line, resulting in a selective increase in IL-8 secretion that induces neutrophil recruitment. This finding corroborates studies that suggest that this parasite uses neutrophils to be phagocytosed by MØs without activating MØ defense mechanisms, thus promoting parasite survival [91,92]. In vitro studies have shown that *L. infantum* EVs also modulate the chemotactic activity of monocytic cells, as well as cytokine production. Therefore, EVs play a modulatory role by promoting immune suppression through the induction of IL-18 and IL-10, facilitating disease establishment [60]. A distinguishing feature of EVs from *L. amazonensis* compared to *L. infantum* and *L. braziliensis* is their ability to highly activate TLR4/TLR2 and induce the nuclear translocation of NF-κB p65. *L. amazonensis* EVs favor a pro-inflammatory immune response, while EVs shed by *L. infantum* and *L. donovani* (viscerotropic species) exhibit a more immunosuppressive profile [90]. Furthermore, EVs’ ability to induce IL-10 production was confirmed by Nogueira and colleagues [90]. EVs derived from *L. guyanensis* and *L. shawi* appear to induce MØs to generate a balanced immune response, generating pro- and anti-inflammatory cytokines at different levels, avoiding an exacerbated infection that, in the case of *L. guyanensis*, can prevent mucocutaneous pathology [67]. On the other hand, EVs derived from *Leishmania* promastigotes released in the sand fly midgut that were then inoculated with *Leishmania* parasites triggered exacerbated reactions [79,93].

Overall, *Leishmania* EVs seem to play a significant role in the establishment of infection in both vertebrate and invertebrate hosts, as well as in disease outcome. Given their ability to modulate the host immune response, EVs hold the potential for the development of novel therapies to treat leishmaniasis or vaccines to prevent these diseases [85].

### 4.2. Host Modulation by Trypanosoma-Derived EVs

Dias-Guerreiro and colleagues investigated the effect that *T. brucei*-derived EVs had on murine MØs and T lymphocytes. Their findings revealed that EVs stimulate MØ activation by overexpressing molecules of the major histocompatibility complex (MHC) and increasing the levels of the CD3 complex along with the FoxP3 nuclear factor in T cells. Therefore, EVs appear to have the capacity to induce a balance between parasite growth and the regulation of the host immune response [94].

Regarding *T. cruzi*-derived EVs, in vivo studies have demonstrated that mice pre-inoculated with EVs and then infected with metacyclic forms of the parasite have shown higher levels of parasitemia; developed a pro-inflammatory response with TNF-α, IL-12, and IL-6 production; and exhibited a more severe pathology than the control mice, associated with a higher mortality rate [78,95,96]. Wyllie and Ramirez [89] stated that EVs containing GP82, a surface glycoprotein of *T. cruzi* that plays a crucial role in parasite adhesion to mammalian cells, can trigger a transient increase in intracellular Ca^2+^, which facilitates cell invasion by *T. cruzi*.

Although EVs are naturally released by all *Trypanosoma* morphologic forms, their specific role in parasite development within the vector remains largely unclear. However, according to Torrecilhas and colleagues [82], epimastigote-derived EVs within the triatomine vector delay parasite transit through the intestinal tract, thereby interfering with the *T. cruzi* life cycle.

## 5. EVs Implicated in Trypanosomatid Evolution by Horizontal Gene Transfer

Horizontal Gene Transfer (HGT) is a non-sexual process through which genetic material is transferred from the genome of one organism to another. This transfer can involve both DNA and RNA, which may replace genetic sequences or integrate new genetic information into the genome of the recipient cell [97]. HGT can facilitate DNA recombination, thereby increasing genetic variation. This natural phenomenon is well characterized across several bacterial species and plays a pivotal role in modulating the interspecific exchange of mobile genetic elements (MGEs), comprising transposons, integrons, and gene cassettes. For example, the horizontal transfer of integrons is recognized as the most impactful in disseminating and diffusing antibiotic resistance genes [98]. Although HGT is common among Archaea and bacteria groups, these organisms can donate their genetic features to other life forms that act as recipients, such as fungi, plants, and animals [99]. When eukaryote–eukaryote HGT is involved, genetic interactions become more complex, as eukaryotes are multicellular organisms with complex metabolic pathways and structural barriers. A parasitic plant transfers mitochondrial genetic material (mtDNA) into the mitochondria of the host plant, as seen in *Amborella*, which has acquired 26 foreign genes from a diverse array of donors over time, including mosses and angiosperms [100]. The HGT dominant mechanism among animals and plants occurs through transposons, but recent findings also identified cell-free DNA (cfDNA) and EVs as gene transfer mediators [98]. HGT events mediated by EVs were first described in bacteria and were proven to be effective in gene exchange, enabling not only intraspecific but also interspecific transfer [101,102]. Other experiments have proven that genomic DNA can also be transferred through EVs, specifically the integration of foreign DNA into recipient cells [103,104].

As previously mentioned, EVs facilitate communication from one cell to another through the horizontal transfer of macromolecules. The role of EVs in mediating HGT in trypanosomatids remains a largely unexplored topic. The limited research on this subject reveals a gap in the understanding of its potential implications. However, Douanne and colleagues [105] investigated the role of EVs in HGT among *L. infantum* and *L. major* parasites, focused on the spread of drug resistance genes. By conducting DNA sequencing and comparative analysis, they discovered that the genomic DNA of drug-resistant strains and the DNA found in EVs contained genetic markers associated with resistance. These findings suggest that EVs released from drug-resistant strains contain genes that reflect the parasite phenotype. Furthermore, the exposure to EVs from drug-resistant strains significantly increased the resistance level of the recipient parasites of both cutaneous and visceral species of *Leishmania*. These data highlight the importance of EVs in disseminating drug resistance.

EVs represent a unique way for gene exchange, which differs from traditional gene transfer methods by enclosing genetic molecules within a protective phospholipid bilayer. The clinical importance of the ability of *Leishmania* EVs to modify recipient parasites and confer drug resistance [106] cannot be overstated. It represents a challenge for controlling and treating *Leishmania* infections, as drug resistance can spread efficiently in the absence of drug pressure. HGT through EVs underscores the nature of these parasites and their capacity to rapidly diversify their genome. Moreover, EVs released by drug-resistant parasites can attenuate stress and promote growth while influencing the proteome of recipient host cells [105]. Furthermore, the discovery of HGT among eukaryotic parasites, facilitated by EVs, enables trypanosomatids to adapt quickly to changing environments.

Gaining a fuller perspective on EVs’ biology and their DNA cargo is essential to better understand their impact on normal parasitic biological functions. While research on EV proteins and RNAs has progressed, the study of DNA carried by EVs remains relatively unexplored. The lack of data in this field presents a challenge to understanding the mechanisms involved in loading DNA into EVs. Thus, it is crucial to acquire insights into the kinetics of EV-DNA packaging along with the pattern of EVs released by the parasite’s morphological forms [107]. In addition, it is essential to investigate the signaling cascades following the exposure of naive parasites to EVs from drug-resistant parasites, as well as the enzymatic activity of the altered pathways associated with EVs’ HGT [105,108].

## 6. Potential of TEVs as Emerging Diagnostic Biomarkers

Accurate diagnosis of these neglected parasitic diseases is essential, not only to ensure the best treatment for patients but also to restrain their geographical spread and improve control measures. The development and implementation of diagnostics for neglected diseases caused by trypanosomatids pose several challenges. An effective diagnostic test should ensure the early detection of infection, present high sensitivity and specificity, and accurately stage current infection, especially for sleeping sickness, while being minimally invasive. It should also be able to differentiate between active and past infection in clinically healed or recovering patients who have been treated and do not cross-react with other parasitic diseases [109,110]. The diagnostic tests available for trypanosomatid infections are conventionally classified as either direct or indirect. Direct diagnostic tests rely on the microscopic identification of parasites, while various indirect assays detect parasite infection by analyzing the host’s humoral immune response. Molecular assays employing polymerase chain reaction (PCR)-based techniques to detect small amounts of parasite DNA have also been applied in the diagnosis of these diseases [111,112].

The identification of biomarkers, which are measurable biological indicators of normal or abnormal processes associated with disease stage or therapy response, is crucial for diagnosis and monitoring disease evolution. These biomarkers can include molecules that signal infection, as well as antibodies, enzymes, and hormones, among other biological components [113].

Several biomarkers have been investigated to be applied in the diagnosis of parasitic diseases, including asymptomatic VL and the acute and chronic clinical phases of trypanosomiasis [114,115,116,117,118]. Among these, EVs have attracted the scientific community’s interest as potential biomarkers to be used in the development of novel diagnostic tools due to their molecular cargo and biological importance in the infection process.

Previous studies have shown that *T. cruzi*-derived EVs carry mucin-associated surface proteins (MASPs), which promote the formation of IgG immune complexes, particularly in patients with the gastrointestinal clinical form of Chagas disease, highlighting their potential as biomarkers for disease diagnosis or prognosis [70]. Bautista-López and colleagues [75] further advanced this field by conducting a proteomic analysis of antigens carried by *T. cruzi*-derived EVs to be used in a novel diagnostic method. This study identified the retrotransposon hot spot protein family (RH), which is absent in *Leishmania* spp., as a promising biomarker, reducing the risk of serological test cross-reactivity between leishmaniasis and trypanosomiasis. Due to the phylogenetic similarity of *Leishmania* spp. and *T. cruzi* parasites, serologic cross-reactivity is common in co-endemic areas, representing a significant challenge to the accurate diagnosis of these parasitic diseases [119,120,121]. In addition to having good reactivity with sera from Chagas disease patients, this RH protein did not react with sera from malaria patients, which makes these EVs apparent specific biomarkers for the diagnosis of digestive Chagas disease.

Another promising strategy under investigation is the use of EVs shed by immunological cells following exposure to parasites or immunogenic proteins. In this sense, a 2020 study showed that murine MØs exposed to EVs shed by *L. amazonensis*-infected MØs exhibited a differential expression of inducible nitric oxide synthetase, IL-6, IL-10, and TNF-α [122]. Therefore, EVs derived from *Leishmania*-infected MØs can also be considered as potential biomarkers for leishmaniasis. Furthermore, the microRNA miRNA-146a, which is a strong biomarker candidate for Chagas disease diagnosis, was found to be upregulated in the heart, plasma, and also in MØ-derived EVs during both the acute and chronic phases of *T. cruzi* infection [123]. Altogether, these findings further underscore the potential of TEVs as promising biomarkers across the clinical phases of trypanosomiasis.

Despite ongoing efforts, no EV-related biomarkers are currently available for the diagnosis of these parasitic diseases, highlighting the need for further research in this field.

## 7. Exploiting TEVs as an Innovative Approach to Controlling Trypanosomatid Diseases

Defined as the process of acquiring immune protection against an infectious agent through the administration of a biological preparation, vaccination constitutes one of the most effective measures for disease control. Currently, there is no vaccine available against human leishmaniasis or trypanosomiasis, but a vaccine for CanL is available in Europe, as dogs are considered the main reservoirs of *L. infantum,* which causes zoonotic visceral leishmaniasis. Over the past few decades, four vaccines for CanL have been developed and commercialized, two in Brazil [124,125] and two in Europe [126]. However, three of these vaccines are no longer available. The remaining vaccine available in Europe is Letifend^®^, which provides up to 72% protection against CanL [127].

The discovery that EVs may carry virulence factors that can manipulate the host immune response to evade parasite elimination by inhibiting the secretion of inflammatory cytokines [59] has paved the way for exploring EVs as potential prophylactic or therapeutic vaccines for diseases caused by trypanosomatids.

In 2010, a published study demonstrated that *L. donovani*-derived EVs modulate cytokine expression by increasing IL-10 and inhibiting TNF-α in human monocytes and restricting the expression of IL-10, IL-12p70, and TNF-α in monocyte-derived dendritic cells [38]. Recent studies have reported that exposure to EVs derived from *L. shawi* and *L. guyanensis*, species that causes American cutaneous leishmaniasis, modulate the in vitro immune response of murine MØs, increasing the generation of cytokines and the expression of MHC class I molecules, suggesting a possible antigenic presentation to CD8^+^ T lymphocytes [67]. Furthermore, canine in vitro studies have shown that *L. infantum*- and *L. amazonensis*-derived EVs shape the immune activation of dendritic cells and influence their interaction with natural killer cells [66,128].

Regarding *T. cruzi* infection, two approaches have been explored: the development of a therapeutic vaccine for Chagas disease that prevents the clinical evolution of chronic chagasic cardiomyopathy and the search for a prophylactic vaccine that avoids infection and disease establishment. MASPs, which are carried by EVs derived from *T. cruzi*, have been tested as a potential vaccine candidate. Mice immunized with synthetic MASPs showed an 86% survival rate after a challenge with *T. cruzi* trypomastigotes and reduced parasitic load in the heart, liver, and spleen [129]. Other studies have investigated alternative EV components as prophylactic vaccines, such as the trans-sialidase family [130], although these enzymes have not been successful enough to be pursued further.

Overall, it is well established that TEVs modulate the host immune response in different ways, depending on the parasite species, the cell type analyzed, and, especially, the competence of the immune system. Thus, it is crucial to highlight that the results obtained from in vitro studies are only a preliminary but important indicator of the effects of EVs on the immune response against trypanosomatids. Research involving EVs is expanding in parasitology, emerging as a highly promising field for the development of new strategies to control or even eradicate neglected trypanosomatid diseases. Although there is evidence that *T. brucei*-derived EVs can shape host immune responses, studies involving the use of *T. brucei* EVs are almost nonexistent, being an area where even greater efforts are needed. Furthermore, due to the versatility, stability, and biocompatibility of EVs, several studies have explored the potential of EV-based vaccines in chronic illnesses, oncological diseases, and other infectious diseases [131]. These lines of research may function as a driving force for technological advances that could be extended to other fields, especially neglected parasitic diseases.

## 8. Conclusions and Future Perspectives for TEVs

**Figure 4:**
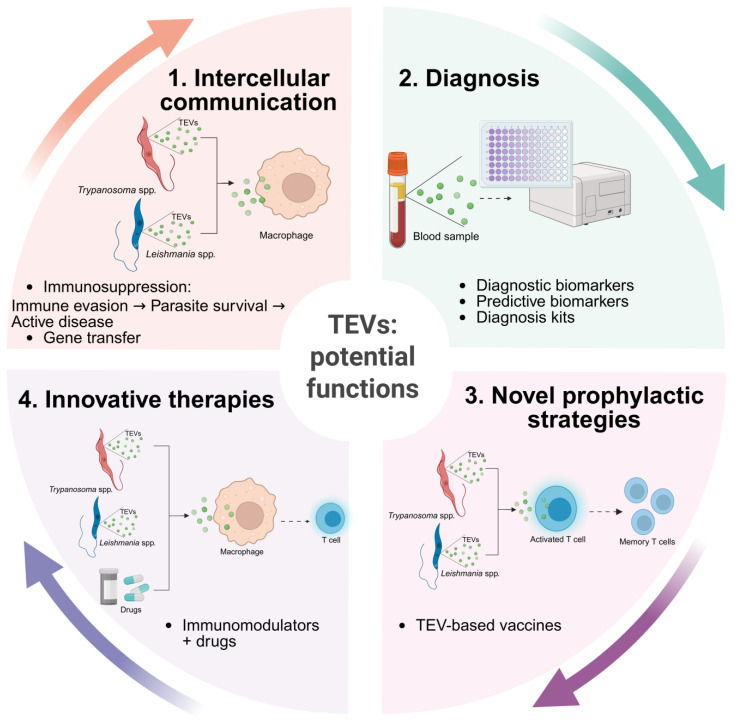
Natural role and potential applications of TEVs. Through intercellular communication (1), TEVs can shape host immune response and mediate gene transfer, thereby influencing parasite biology. Furthermore, TEVs show potential to advance diagnostic approaches (2), promote vaccine development (3), and contribute to innovative therapeutic strategies (4) for diseases caused by trypanosomatids.

The study of EVs in the context of disease caused by trypanosomatids and other NTDs undoubtedly holds great potential for improving our understanding of parasite biology, developing novel diagnostic tools, and creating immunomodulatory targeted therapies and vaccines. EVs play a crucial role in parasite survival, pathogenesis, and host–parasite interactions by mediating the transfer of bioactive molecules such as proteins, lipids, DNA, and RNA to recipient cells. The exploitation of TEVs by the scientific community has potentiated the unraveling of complex host–parasite dynamics and the understanding of the molecular composition and functional roles of TEVs. This may reveal new strategies to control parasite dissemination and disease progression (Figure 4). Future studies should focus on elucidating the molecular mechanisms underlying the biogenesis of TEVs, cargo selection, and their functional impact on host cells. Multi-disciplinary approaches combining proteomics and transcriptomics will be essential to advance the understanding of these parasitic vesicles.

TEVs hold great potential as non-invasive biomarkers for the early and accurate detection of trypanosomatid infections. Their identification in easily accessible biological fluids such as blood, urine, and saliva could revolutionize current diagnostic approaches by offering higher sensitivity and specificity. However, further research is needed to standardize isolation and detection protocols, ensuring reproducibility and scalability for clinical implementation. TEVs carry parasite-derived antigens and can elicit robust immune responses, having the potential to be exploited as immunomodulators in combination with antiparasitic drugs, enhancing therapeutic efficacy while minimizing systemic toxicity. Furthermore, they have the potential to induce protective and long-lasting immunity against trypanosomatid infections. Despite these promising applications, challenges such as immunogenicity, stability, and large-scale production must be addressed to facilitate their translation into effective prophylactic tools.

In conclusion, while significant progress has been made in understanding the role and potential applications of TEVs, several challenges remain to be addressed before their full clinical and immunological potential can be fulfilled. Thus, key issues include ensuring the purity, safety, and reproducibility of TEVs, as well as their standardization. Ongoing TEV research efforts, technological advances, and collaborative initiatives will be crucial in unlocking new opportunities to combat NTDs, particularly trypanosomatid-associated diseases. These efforts will not only improve global health for animal and human populations in the One Health approach but also contribute to societal advancement and economic stability.

## Abbreviations

The following abbreviations are used in this manuscript:

NTDs	Neglected tropical diseases
NZDs	Neglected zoonotic diseases
VL	Visceral leishmaniasis
CL	Cutaneous leishmaniasis
CanL	Canine leishmaniasis
EV	Extracellular vesicle
ISEV	International Society for Extracellular Vesicles
MISEV	Minimal Information for Studies of Extracellular Vesicles
TEVs	Extracellular vesicles from trypanosomatids
MØ	Macrophage
HSP	Heat shock protein
TRYPI	Tryparedoxin peroxidase
LACK	Activated protein kinase c receptor
STI 1	Stress-induced protein 1
SRA	Serum-resistance-associated protein
FCaBP	Flagellum calcium-binding protein
SAPA	Shed acute-phase antigen
TLR	Toll-like receptor
TcTASV-C	Trypomastigote Alanine-, Valine-, and Serine-rich proteins
PPG	Proteophosphoglycan
SAP	Secreted acid phosphatase
TF	Transcription factor
mTOR	Mammalian target of rapamycin
PTP	Protein tyrosine phosphatase
NO	Nitric oxide
HGT	Horizontal Gene Transfer
PCR	Polymerase chain reaction
MASP	Mucin-associated surface protein
RH	Retrotransposon hot spot protein family
